# Literature Review on Conjugated Polymers as Light-Sensitive Materials for Photovoltaic and Light-Emitting Devices in Photonic Biomaterial Applications

**DOI:** 10.3390/polym16101407

**Published:** 2024-05-15

**Authors:** Paolo Coghi, Carmine Coluccini

**Affiliations:** 1 Laboratory for Drug Discovery from Natural Resources & Industrialization, School of Pharmacy, Macau University of Science and Technology, Macau 999078, China; coghips@must.edu.mo; 2 Institute of New Drug Development, College of Medicine, China Medical University, No. 91 Hsueh-Shih Road, Taichung 40402, Taiwan

**Keywords:** conjugated polymers, photonic devices, photonic biomaterials

## Abstract

Due to their extended p-orbital delocalization, conjugated polymers absorb light in the range of visible–NIR frequencies. We attempt to exploit this property to create materials that compete with inorganic semiconductors in photovoltaic and light-emitting materials. Beyond competing for applications in photonic devices, organic conjugated compounds, polymers, and small molecules have also been extended to biomedical applications like phototherapy and biodetection. Recent research on conjugated polymers has focused on bioapplications based on the absorbed light energy conversions in electric impulses, chemical energy, heat, and light emission. In this review, we describe the working principles of those photonic devices that have been applied and researched in the field of biomaterials.

## 1. Introduction

**Figure 1:**
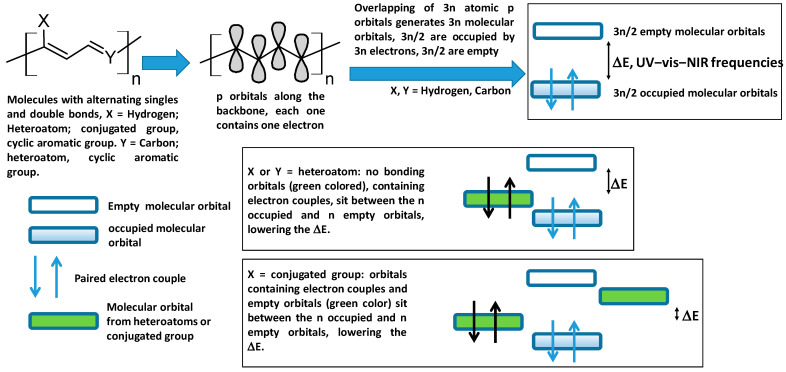
Structure and electronic properties of conjugated polymers.

Along their molecular backbone, conjugated organic molecules exhibit alternating single and double bonds, or π-conjugation. Conjugated polymers are defined as polymers of conjugated molecules that show extended π-conjugation throughout the entire skeleton. Their structure and electronic properties are shown in Figure 1 [1]. This structural feature of molecules is caused by the overlapping of atomic p orbitals, which results in frontier molecular orbitals that are delocalized along the molecular skeleton and are energetically close to one another. These molecules absorb energies corresponding to UV–vis–near IR light frequencies, which causes electrons to move from the highest occupied molecular orbital (HOMO) to the lowest unoccupied molecular orbital (LUMO). As a result, these molecules exhibit high electron mobility along the molecular backbone. The precise frequency depends on the molecular structure and atomic composition. Because conjugated polymers have more atoms and electrons than their counterparts in small molecules, the effects of electronic delocalization are more pronounced. Monomers are conjugated units that often exhibit a cyclic/polycyclic structure that imparts rigidity and, consequently, more efficient p orbital overlapping; further conjugation also affects amplification.

**Figure 2:**
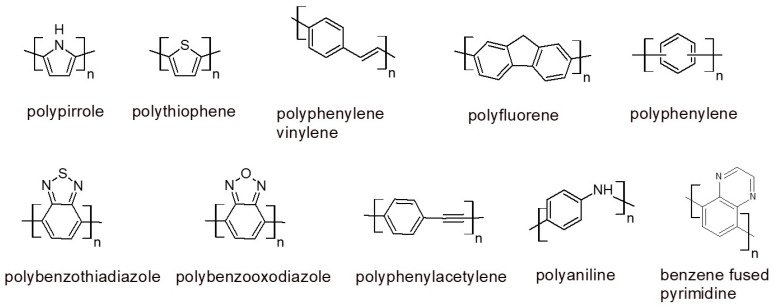
Fundamental structures of conjugated polymers utilized as photonic materials. We have analyzed the structure described in the literature about the development of conjugated polymers [1,2,4], and we understood that all structures are obtained by modification (addition of an akyl chain, copolymerization with other monomer) of the structure shown in the figure.

Conjugated polymers exhibit the greatest optical properties (absorption and fluorescence intensity, photostability), due to having the greatest amount of π-conjugation. Photonic devices consist of machines that convert part of the absorbed light energy into different kinds of energy like electrical, thermal, chemical, and light emission [2]. The first generation of such devices exploited light-absorbing inorganic semiconducting materials; research is currently focused on organic conjugated compounds that show easier fabrication and processability, easier structural modification for varying the optical properties, less toxicity, suitability for solution-based processing techniques on flexible substrates, and the lowest fabrication costs [3]. In conjugated compounds, photonic property tuning can be achieved using molecular structure and functional group variations (synthetic tunability). The structures in Figure 2 represent the fundamental conjugated polymers utilized in photonic devices and biomaterials. More complex structures are obtained by connecting alkyl or hetero-alkyl chains, conjugated electron donor or accepting groups, and fused heterocycles by copolymerizing two of the monomers shown in the figure [4].

In this review, we describe the working principles of photonic devices, how conjugated polymers are utilized, and how scientific researchers are currently trying to utilize these principles in photonic biomaterials. The photonic devices described in this manuscript are the devices that exploit light absorption (photovoltaic, PV) and emission (organic light-emitting devices, OLEDs). The PV vehicle separately photo-generates electrons and holes for generating voltage and current in a circuit. Research of photonic biomaterials has investigated how electron and hole photoseparation can be potentially exploited for medical therapies. The OLED converts the absorbed light into an emission that can be applied in bioanalyte detection.

## 2. Photovoltaic Effect and Devices

### 2.1. History of Photovoltaic Effect

**Figure 3:**
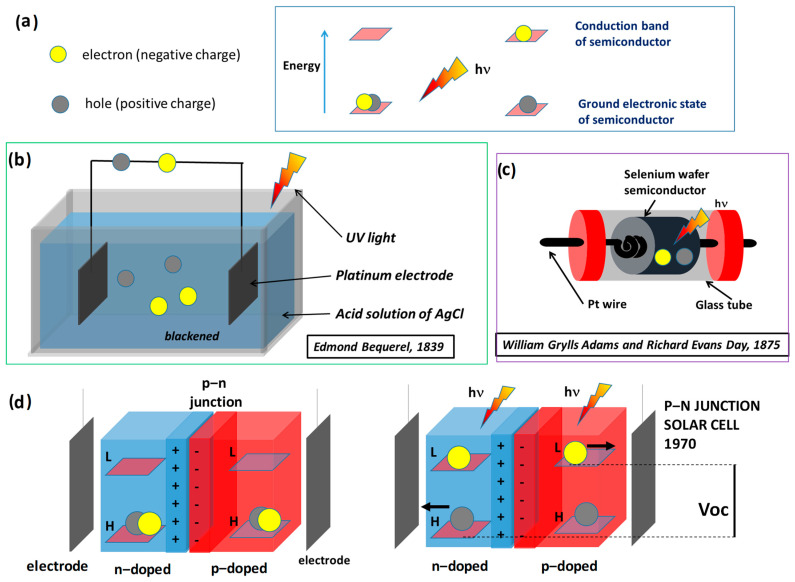
Photovoltaic effect and devices working principle depicted according to the descriptions in the specific publications [5,6]: (**a**) principle of photo-induced charge separation; (**b**) photo-induced conductivity of solutions; (**c**) photo-induced conductivity of solid selenium devices; and (**d**) description of p–n junction solar cells.

The photovoltaic effect takes advantage of the fact that when a semiconductor absorbs light, free electrons and holes are generated. Consequently, a weak current is measurable between electrodes that are placed in contact with the semiconductor, as demonstrated experimentally in the seventeenth century and shown in Figure 3a–c. Photovoltaic development has spanned almost two centuries [5,6]. In 1839, the French physicist E. Becquerel noted that using UV light to illuminate solutions containing a metal halide salt produced a current between two platinum electrodes immersed in the electrolyte. This phenomenon was observed in a solid by Adams and Day during the second half of the nineteenth century. Two platinum electrodes were placed at the extremities of a selenium bar, and a voltage between the electrodes was observed when UV light was harvested on the selenium. At the end of the nineteenth century, Moser observed that the sensitization of silver salts with dye allowed for the exploitation of visible and infrared light for current production. Photovoltaic technology aims to efficiently separate photo-generated free electrons and holes to avoid their recombination and to transport the two different charges toward two different electrodes and create a stronger voltage. In photovoltaic devices, two semiconductors are placed next to each other; one works as an electron carrier, while the other works as a hole carrier. Each one is connected to one electrode. Commercial photovoltaic devices utilize n-doped (electron-enriched) silicon and π-doped (hole-enriched) silicon, as shown in Figure 3d. When the two materials are placed next to each other, an electric dipole is generated at the interface junction because the electrons of the n-material are attracted by the holes of the p-material. The negative charges are localized on the p-side of the interface, and positive charges are localized on the n-side. The dipole at the junction drives the photo-generated free electrons toward the n-silicon (the electron carrier) and the free holes toward the p-silicon (the hole carrier). A potential is generated between the electrodes that are in contact with the two semiconductors. In 1941, the first p–n junction photovoltaic device was produced, and this technology evolved significantly after 1970.

Although device performance allows for domestic utilization, the drawback of this technology is the cost of the materials. The search for cheaper light absorbers is desirable in order to broaden the utilization of photovoltaic devices. Researchers are developing two new technologies for overcoming the shortcomings of traditional devices: fully organic solar cells and dye-sensitized solar cells.

### 2.2. Fully Organic Solar Cells

**Figure 4:**
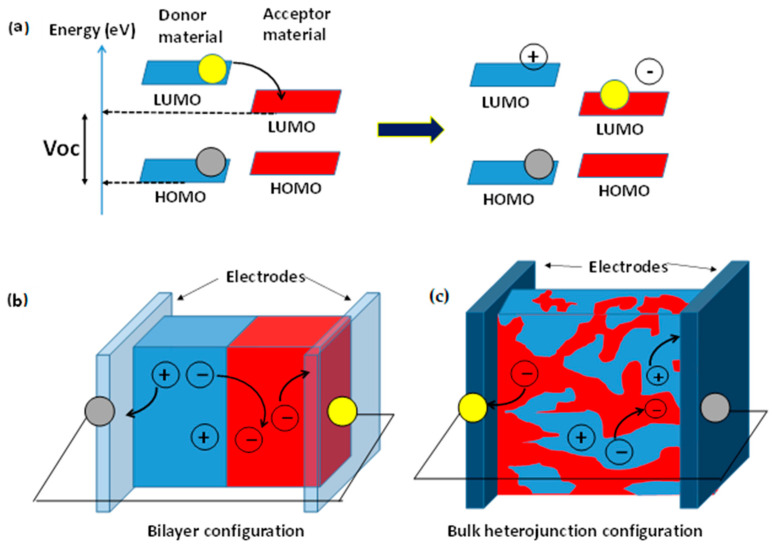
Description of fully organic solar cells according to the descriptions of the specific publications about these devices [2,4,6,7]. (**a**) Principle of charge transfer after light absorption. Open circuit voltage, Voc, is the difference between the LUMO of the accepting material and the HOMO of the donor material. (**b**) OPV bilayer configuration. (**c**) OPV bulk heterojunction configuration.

As shown in Figure 4, two organic semiconductors—an electron donor and an electron acceptor—are placed next to each other. As a consequence of light absorption, the donor injects electrons to the acceptor through the interface, and the acceptor injects holes to the electron donor. A potential is generated between the electrodes that are connected to the semiconductors. The electron donor material carries the holes to the electrode, and the electron acceptor material carries the electrons. The organic semiconductors can be polymers or small molecules [7].

**Figure 5:**
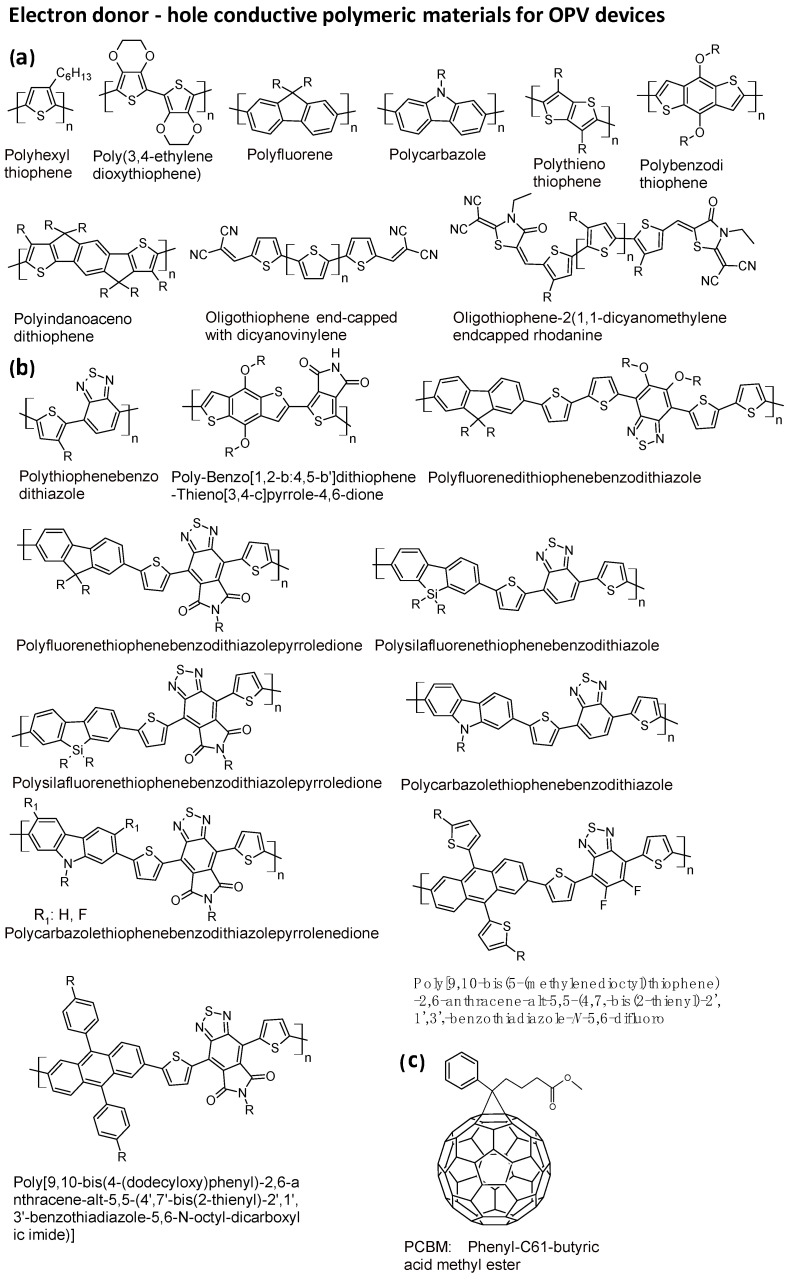
Representative electron donor–hole conductive polymeric structure materials for OPV devices used in combination with electron acceptor PCBM (phenyl-C61-butyric acid methyl ester), obtained the following [6]: (**a**) Traditional π-conjugated polymers obtained by polymerization of electron-rich conjugated monomers based on thiophene, fluorene, carbazole, thienothiophene, benzothiophene, indanocenethiophene, and benzodithiophenethienopyrroledione. (**b**) –DA- π-conjugated polymers obtained by linking electron donor π-conjugated monomers like thiophene, fluorene, silafluorene, carbazole, and antracene with electron acceptors like benzodithiazole and benzodithazolepyrroledione. (**c**) Structure of PCBM, electron-poor molecule, the most used electron transporter in OPV devices. R are alkyl groups.

Conjugated polymers have been widely studied as potential organic semiconductors since the 1970s, and the first solar cell based on poly(hexylthiophene) was produced in 1984 [8]. The first evidence regarding charge photo-generation in a phthalocyanine in the presence of an electron acceptor material dates back to 1980; a few years later, the first results regarding donor–acceptor planar heterojunction cells were published. The concept of the bulk heterojunction was introduced in 1995 [8]. Almost simultaneously, research on OPVs based on polymers and small molecules began. In these first years, donor polymeric materials within devices exhibited the best performance and were more widely studied (Figure 5a, polymers of π-donor conjugated molecules) [6]. In 2005, the donor material poly(3-hexylthiophene) (P3HT), coupled with PCBM (phenyl-C61-butyric acid methyl ester), achieved a record efficiency of 5%. Successively, low-band-gap polymers, in which the donor and acceptor groups are alternated (structure: -D-A-D-A-), surpassed the performances of poly(3-hexylthiophene). Over the last five years, BHJ solar cells, based on low-band-gap polymers (benzo[1,2-b:4,5-b′]dithiophene–thieno[3,4-c]pyrrole-4,6-dione polymer) and coupled with PCBM, have achieved an efficiency of 9% [8]. The design of the optimal p type polymer still now constitutes the main challenge to overcome the Coulombic attraction hole-electron that must be separated into free charges in the interfacial region, and in recent years, several polymers have been prepared and tested [2,6].

As donor materials in OPVs, due to hyperconjugation, conjugated polymers offer the advantages of high absorption intensities and a wide range of absorption frequencies. The drawbacks of these polymers relate to the low reproducibility of their synthesis and purification and, hence, their electronic properties. The crude material contains conjugated chains of various lengths and requires fractionating with different solvents to reduce polydispersity. Furthermore, it is often necessary to remove remnant terminal groups using appropriate chemical treatments. These additional treatments increase the costs and environmental impact of producing the material. The synthesis and purification of small molecules are more reproducible. Furthermore, small molecules are more soluble in organic solvents and are more easily processed for blend fabrication. In 2000–2001, results regarding the first BHJ based on small molecules were published. The donor material was phthalocyanine or benzocoronene, and the acceptor was perylene. The performances of the devices were very limited, in the order of 0.3%. In 2006, interest in small molecules was re-piqued [9]. Different types of donor small molecules were synthesized and tested. PCBM was utilized as an acceptor material. The linear oligothiophenes reached efficiencies in the order of 0.5%. Many donor functional groups were functionalized for the synthesis of dyes: oligothiophenes; triphenylamines; diketo-pyrroles; boron-dipyrromethenes; squaraines; and dicyanopyrene derivatives.

After the first results, it was clear that a molecular design with only a strong donor molecular group as a photoactive unit did not allow for high efficiency. This is due to the poor absorption in terms of visible and infrared light; although the photovoltage was higher than in polymer-based devices, the currents were very low. The linear or dendritic oligothiophenes reached efficiencies in the order of 1.5%. The insertion of acceptor groups into the molecular design increased the visible and IR absorption and also increased the efficiency. Oligothiophenes, end-capped with acceptor groups (Figure 5a last two structures), like dicyanovinylene, allowed for efficiencies in the order of 5.0%. An oligothiophene-like small molecule with seven conjugation units as the backbone and 2-(1,1-dicyanomethylene)rhodanine as the terminal unit allowed for the fabrication of devices with efficiencies over 9% [10].

Push–pull molecules based on triphenylamine reached efficiencies of 6.7%. DADADA oligomers, where D (donor) is thiophene and A (acceptor) is benzothiadiazole, reached efficiencies of 8% (Figure 5b, first structure) [8,9]. It was understood that the -DADA- sequence is the bedrock for designing highly efficient donor materials. For this reason, -DA- polymers started to be designed, prepared, and tested in devices with PCBM. The first highly efficient -D-A-D-A- polymer was benzo[1,2-b:4,5-b′]dithiophene–thieno[3,4-c]pyrrole-4,6-dione (Figure 5b, second structure), as previously mentioned. Donor–acceptor sequenced polymers still now constitute the main design of polymers for OPV (Figure 5b, structures of the most performant polymers), and the choice of the suitable donor and acceptor monomers allows for the optimization of the HOMO-LUMO bandgaps for the most efficient electron transfer towards the electron acceptor PCBM and highest voltage [6]. The most studied donor–acceptor structures (the most representative shown in Figure 5b) consisted in donor units like thiophene, fluorene, benzothiadiazole, silafluorenes, carbazoles, and anthracenes and acceptors like modified benzodithiazole. The best device performances reach efficiencies around 10% [6].

The most utilized electron acceptor material is the PCBM consisting of a fullerene linked to a phenyl ring and butyric acid methylester (Figure 5c). Fullerene’s structure consists of sp^2^ carbons arranged in a polyhedral spherical-shaped closed cage, stabilized by electron injection in the antibonding LUMO orbitals; for this reason, this molecular unit is electron withdrawing [11].

Researchers are also trying to find an alternative to using PCBM as an acceptor material. PCBM is a good electron carrier and is easy to process in order to fabricate blends. The drawback is that it absorbs light very weakly and does not contribute to the current. Acceptor molecules that absorb visible light can contribute to the donor current. Low-band-gap molecules can also be valid candidates as acceptor materials. Different linear donor–acceptor molecules are under investigation to replace PCBM [12,13].

**Figure 6:**
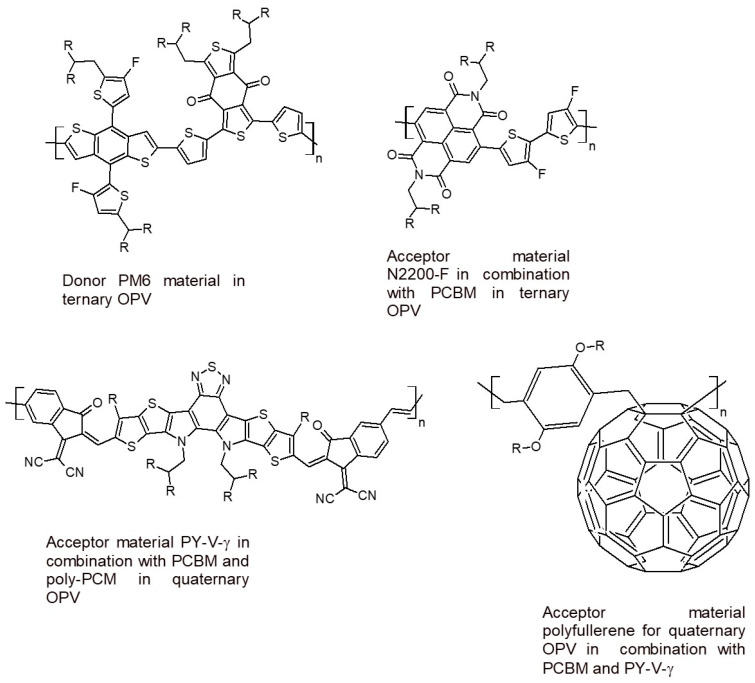
Conjugated polymers for ternary and quaternary OPVs with high performances [12,13,14].

The highest power conversion efficiency can be achieved in ternary OPV configuration devices, due to the improved short-circuit current density (Jsc) and fill factor (FF). In these devices, a combination of a conjugated polymeric light absorber with a fullerene-based material is used as an electron acceptor material. The former broadens the absorption spectrum to increase the short-circuit current density (Jsc); the latter finely tunes the blend morphology and charge transport [14]. In 2020, a device was reported that used a fullerene derivative (PC71BM in Figure 6) with a non-fullerene acceptor (N2200-F in Figure 6) blended with a polymer donor (PM6 in Figure 6), allowing for an efficiency of 8.11%. Recently, a high-performance quaternary OPV with an efficiency of 16.9% was fabricated by using the same donor (PM6) and a combination of polyfullerene, PCBM, and a conjugated polymer (PY-V-γ in Figure 6) as an acceptor material [15].

### 2.3. Dye-Sensitized Solar Cells (DSSCs)

**Figure 7:**
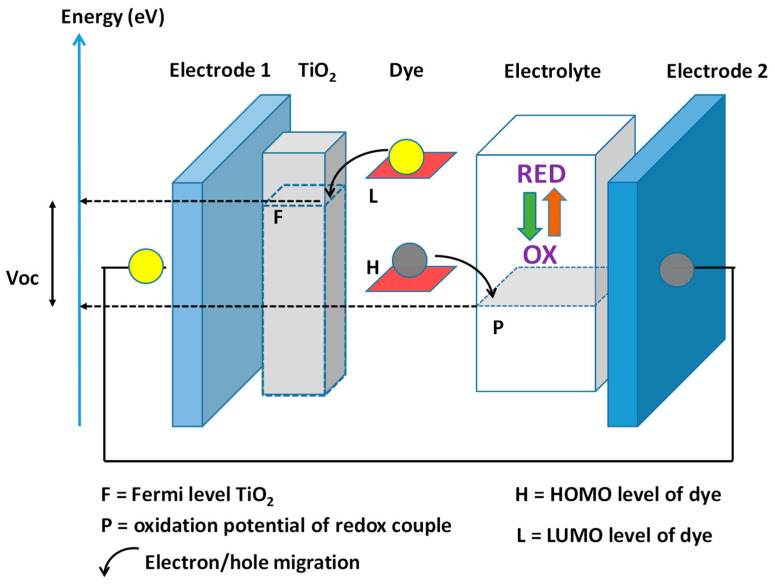
Dye-sensitized solar cell scheme according to the descriptions of the literature data [16,17,18,19,20,21,22,23,24]. The dye absorbed on TiO_2_, under light absorption, injects electrons into TiO_2_ and then to electrode 1; it injects holes into the redox material and then to electrode 2. The energy difference between the oxidation potential and the Fermi level of TiO_2_ is the open circuit voltage, Voc.

The working mechanism of dye-sensitized solar cells is shown in Figure 7. A sensitizer (a dye that absorbs light) is absorbed in titanium dioxide (TiO_2_), which works as an electron transporter, and the hole transporter is a redox material. The sensitizer absorbs light and injects electrons into the TiO_2_ and holes to the redox material, which becomes oxidized. The electron migration between the two electrodes reduces the redox material, restoring the initial configuration [5,7,16].

The first oxide semiconductors to be sensitized were ZnO and SnS_2_ [17,18]. Successively, efforts were made in sensitizing TiO_2_, due to the material’s easy availability [19,20]. Graetzel and coworkers started to produce the first regenerative cells [21], and after a few years, they obtained efficiencies of 7% [22]. A ruthenium complex was used as the sensitizer, and a redox material (I^−^/I_3_^−^) was used as the hole transporter. The best performances were obtained in 1993 when the dye N719 was utilized [23]. The compound is a ruthenium complex, where the ligands are two bipyridines with carboxy groups in positions 4 and 4′ and two SCN^−^s. The function of the carboxy functional groups is to bind the TiO_2_. The efficiency of the devices was 12%. N719 is commercial and is utilized as a reference, although different ruthenium complexes were studied and tested as sensitizers [24]. In 2011, the efficiency of N719 was matched with a porphyrin dye and a cobalt complex as the redox couple [25]. The utilization of perovskites as sensitizers is very promising. After the work of Miyasaka and others in 2009, the power conversion efficiency of dye-sensitized solar cells rapidly increased from 3.8% to 15% in the space of four years [26,27]. Two drawbacks of these materials are their low availability and their toxicity. Fully organic dyes attracted the attention of researchers because of their low toxicity and low cost, due to the absence of heavy metal atoms [16,28,29,30]. The structure is of the D-π-A type, where D is an electron donor group that is connected to an acceptor group A through a π-bridge. The electron acceptor must bind the TiO_2_. The dye, in the ground state, exhibits more electron density on the donor group. In the excited state, the electron density is transferred to the acceptor group. When the dye is absorbed on TiO_2_, the electrons are injected into the titanium oxide. Many donor groups have been exploited: coumarins, indolines, tetrahydroquinolines, carbazoles, dialkyl-anilines, and triphenylamines [16,28,29,30]. The donor group that has been most widely utilized is triphenylamine, which is easily functionalized and confers high absorption coefficients and good stability [31]. Coumarins, although possessing high absorption coefficients, self-associate on the surface of the semiconductor, and few molecules are absorbed. Indolines allow for high efficiencies of almost 9% but are not very stable. The other donor groups confer low efficiencies. As an acceptor group, cyanoacrylate is the most utilized, due to the electron injection efficiency in the metal oxide. The donor group, directly connected to the acceptor group, injects electrons into the semiconductor. However, the insertion of a π-bridge in between moves the absorption to higher wavelengths and strongly increases the efficiency. As an example, triphenylamine, when simply connected to a cyanoacrylic group, confers an efficiency of 3% to the cell [32]. The insertion of a phenyl-vinylene bridge increases the efficiency to 9.1% [33]. The dye C217, where a di-alkoxy triphenylamine is connected to a cyanoacrylic acceptor through an EDOT-thienothiophene bridge, confers to the cells an efficiency of 9.8%; for many years, this was the record efficiency for full organic dyes [34]. Although they have high intensity, a drawback of organic dyes is their narrow wavelength absorption window. A solution to this problem is the contemporaneous absorption on TiO_2_ of more dyes that absorb at different frequencies. In 2017, the utilization of a couple of fully organic dyes (derivatives of triphenylamine that were previously utilized but only singularly) allowed us to obtain a cell with an efficiency of 11%. A complex of Cu, an inexpensive solid, was utilized as a hole conductor. The efficiency under diffuse light was 28% [35].

### 2.4. Conjugated Polymers as Hole Transporters in Dye-Sensitized Solar Cells

**Figure 8:**
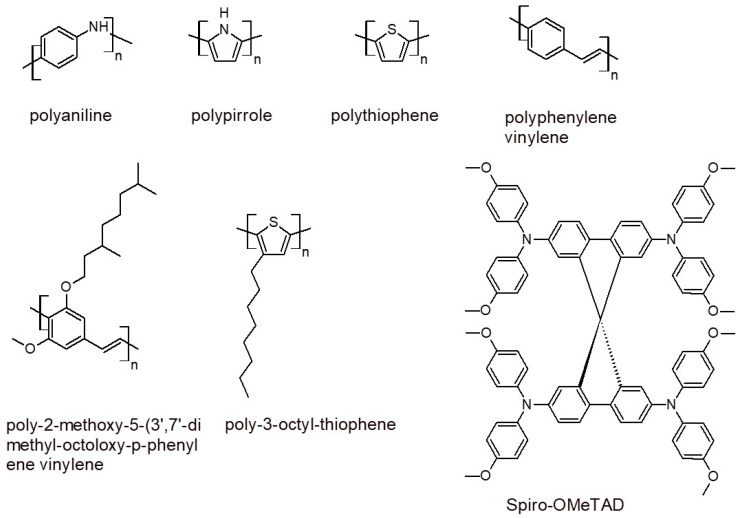
Conjugated polymers studied as electrolytes for DSSCs as described in the literature [36].

The iodide (I^−^)–triiodide (I_3_^−^) redox couple (derived from I^−^ and I_2_) is the most used charge mediator in DSSCs due to it having the most suitable redox potential for reducing oxidized dyes. This redox couple absorbs visible light, and at high concentrations, it can affect the light absorption of the dye. I_3_^−^ ions can react with injected electrons, and high concentrations increase the dark current. For this reason, the concentration of I^−^/I_3_^−^ must be optimized [36]. We must also mention that the same dyes under investigation are not efficiently oxidized by this liquid electrolyte. Not only are scientists considering new redox couples with different redox potentials, they are also considering hole transporter materials; in particular, the conjugated polymers of some of these are shown in Figure 7 [36]. The utility of these polymers consists in retaining the mechanical properties of organic compounds while displaying the conductivity of the metals. The energy conversion is low in comparison to the junctions based on inorganic materials; however, the energy conversion is far from the theoretical limit, and this means that the material cannot compete with traditional inorganic electrolytes. This is fundamental to the conducting polymer’s wettability. Polymers cast from solutions must penetrate the TiO_2_ nanoparticle pores, maintaining good contact with the adsorbed sensitizer. The polymeric material must be a poor light absorber in the dye absorption spectral range. The valence band of the p-type conducting polymer must be higher than the dye’s energy ground state to avoid a dark current and to allow for hole transfer from the TiO_2_ to the conjugated polymer. The hole mobility in the conjugated polymer must be sufficiently high to prevent charge recombination. The difference between the dye’s ground state and the valence state of the polymer is the open-circuit voltage (VOC) of the device. In any case, the efficiency of cells with these polymers is around 1%; these low values are due to the efficient TiO_2_ nanoparticle filling and the poor electronic contact between the dye molecules and the hole conductor. Conjugated polymers with higher wettability, like the highly conjugated triphenylamine cyclo-oligomer Spiro-OMeTAD, shown in Figure 8, displayed better efficiencies of around 2.5%.

## 3. Biomedical Applications for Charge Photo-Carriers

**Figure 9:**
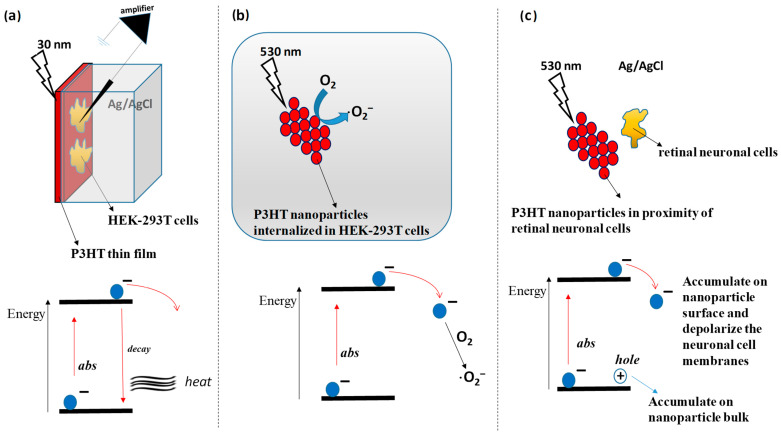
Exploitation of photovoltaic phenomenon in biomaterials as described in the literature [37,38,39]. (**a**) HEK cells cultured on poly-3-hexylthiophene (P3HT) thin film induce activation of vanilloid receptor 1 when exposed to 530 nm light through local heating and solution acidification (abs means absorption) [37]. (**b**) Irradiation of poly-3-hexylthiophene (P3HT) nanoparticles internalized in HEK-293T photo-catalyzes the formation of radical oxygen (·O_2_^−^) from oxygen (O_2_) that elicits intracellular Ca^2+^ dynamics [38]. (**c**) Irradiation of a poly-3-hexylthiophene (P3HT) nanoparticle induces the accumulation of negative charge at the nanoparticle surface that depolarizes the membrane of the vicinal retinal neuronal cells [39].

In photo-electrical devices, the problem of poor efficiency strongly limits the application of conjugated organic molecules. The biocompatibility of organic molecules makes them very promising in biomedical applications, and in the case of organic semiconductors, they can offer opportunities for charge photo-generation and photo-transportation in biological environments. Many conjugated organic molecules absorb UV light that easily penetrates biological tissues, and as a consequence of this, they can transfer charges or heat to the surrounding cells. A polymer, upon light irradiation, can transfer excited electrons directly to the biological environment, can transfer electrons to an acceptor material carrying positive charges (electronic holes) to the surrounding environment, and can decay energetically emitted heat, as shown in Figure 9a [37]. The UV–vis irradiation of primary hippocampal neurons excises retinal tissues, and epileptic brain slices grown on poly-3-hexylthiophene-coated glass substrates induce transient depolarization followed by sustained hyperpolarization, due to a local temperature increase followed by intense light absorption by the polymer; this affects the cell’s membrane capacitance and the electron chemical equilibrium potential [38]. As shown in Figure 9a, upon UV–vis light irradiation, a thin poly-3-hexylthiophene (P3HT) film deposited by spin-coating on top of a glass substrate was demonstrated to activate vanilloid receptor 1 (a kind of transient receptor potential channel; TRP) in HEK-293T cells. In this case, the effect was due to the acidification of the extracellular bath and the increase in the temperature [39]. A P3HT thin film also activates retinal neuronal cells and restores sensitivity in blind rats [40]. After being exposed to light and internalized in an HEK-293 cell, poly-3-hexylthiophene nanoparticles irradiated in an electrolytic solution generated a photocurrent and elicited intracellular Ca^2+^ dynamics [37]. The production of reactive oxygen through photocatalysis explains this phenomenon, seen in Figure 9b. In animal models of dystrophic retinal neuronal cells, interfaced poly-3-hexylthiophene nanoparticles cause intermembrane depolarization, which, in turn, restores visual responses, seen in Figure 9c [38]. This phenomenon can be explained by assuming that the polymer photoexcitation causes an accumulation of negative charges on the nanoparticle surface near the neuronal membranes and of positive charges in the bulk.

**Figure 10:**
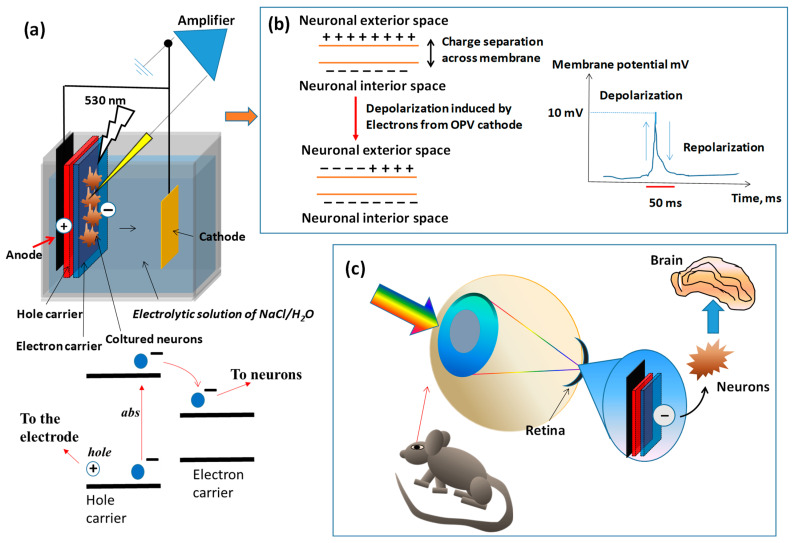
Photovoltaic devices as neuronal cell activators in vitro and for retina replacement in an animal model; we drew the device and we described the concept according to the description of the published articles about this topic. (**a**) Device description according to the literature [39]. The bilayer OPV cell placed in an electrolytic solution, with the hole carrier material in proximity of an electrode, and neurons cultured on the electron carrier surface. Light induces charge separation at the bilayer interface and electrons are injected in the electron carrier material. (**b**) Such electrons are also in proximity of the cultured neuron membrane and induce the neutralization of the across-membrane charge separation (depolarization) with a maximum of 10 mV membrane potential. The depolarization will be transferred to the neighbor neurons after 50 ms (repolarization). (**c**) The depolarization through the neuron is transferred to the brain cell designated for vision [40,41]. For this reason, researchers tested the OPV as a replacement for a damaged retina in an animal model.

Tandem systems consisting of a conjugated polymer hole conductor and an electron conductor material can also be used for activating neuronal signals [41]. Upon light irradiation, poly-3-hexylthiophene injects electrons into the electron-conducting material (PCBM or PEDOT) that interacts with cell membranes and depolarizes them. These photovoltaic devices have been tested for retina replacement in animal models (Figure 10) [42,43].

### Polymers and Infectious Disease

**Figure 11:**
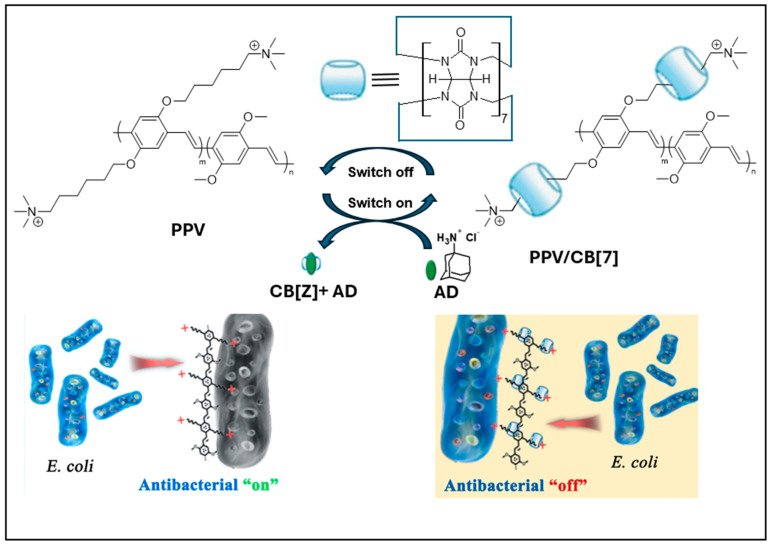
Some water-soluble conjugated polymers. PPV supramolecular antibacterial system. Reprinted, in part, with permission from Ref. [48].

Bacterial infections continue to pose a substantial threat to global health, persisting despite the extensive utilization of various antibiotics over decades and ongoing endeavors by researchers to unearth novel antibiotic solutions. Strategies based on nanomaterials, especially those not dependent on traditional small-molecule antibiotics, show potential, largely because they can bypass the mechanisms employed by drug-resistant bacteria. Consequently, the utilization of nanomaterial-based formulations has garnered interest in the realm of antibiotic therapy [44,45]. With advancements in optical technology and the emergence of novel photosensitizers, photodynamic antibacterial therapy (PDAT) has emerged as one of the most promising approaches for combating infections caused by drug-resistant bacteria. Conjugated polymer nanomaterials have been widely used in the killing and detection of pathogens [46,47,48]. In particular, water-soluble conjugated polymers, as shown in Figure 11, have been applied extensively in the realm of photodynamic therapy for combating pathogenic bacteria, owing to their remarkable ability to generate reactive oxygen species (ROS) in the presence of oxygen [49].

## 4. Light-Emitting Materials

### 4.1. Mechanistic Aspects of Light Emission

**Figure 12:**
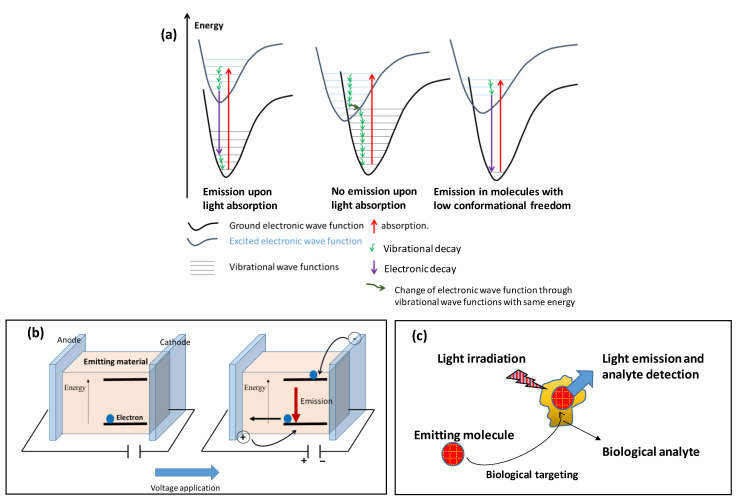
Emission mechanism and its exploitation in photonic devices, as it is described in the literature [50,51,52,53,54]. (**a**) Description of emission through electronic wave function diagrams. Emission always arises from molecules where the excited and ground electronic states’ energies are not overlapped (α). When excited and ground electronic states partially overlap their energetic state and exhibit some vibrational states with the same energy, they will not exhibit emission because the transition excited–ground state will take place with no energy changes (β). Molecules where excited and ground states are energetically partially overlapped, but they do not exhibit vibrational states with the same energy, are highly probable in compounds with low conformational freedom, and the molecule emits light (γ). (**b**) OLED working principle. The emitting material is inserted between two electrodes. The application of voltage attracts the electrons of the HOMO orbital to the anode, and injected electrons into the LUMO orbital from the cathode. The migration from LUMO to HOMO is the step that determines the emission. (**c**) Emitting materials for biomolecule detection.

A molecule that absorbs light can partially release the absorbed energy with light emission, as shown in Figure 12. The energy decays through vibrational states (or wave functions) inside the same electronic state (or wave function) do not generate emissions. The energetic decay, from an electronically excited state to an electronic ground state, generates emissions when the two electronic wave functions exhibit no vibrational states with the same energy (Figure 12a, situation α). The excited electronic state and the ground electronic state of a molecule can exhibit some vibrational states with the same energy (Figure 12a, situation β). When the electronic state changes through vibrational states with the same energy, no emission is observed. A molecule with few vibrational states exhibits a low probability that vibrational wave functions of different electronic states will overlap energetically (Figure 12a, situation γ) and has the highest probability of emitting light. The molecular aggregation strongly influences the vibrational states and, as a consequence, the light emission changes. When designing performant emitters, one must consider the electronic energetic states as well as the shape of the molecules that influence the roto-vibrational energies.

### 4.2. Inorganic and Organic Emitter Applications

The invention of light-emitting materials has revolutionized display and lighting source technology. Electro-optic devices and sensors for analytical devices are composed of light-emitting materials. Regarding the materials used to manufacture electro-optic devices, light-emitting diodes (LEDs) represent an efficient long-life illumination system with a significant impact on energy savings [50]. Currently, commercial LED devices are mainly composed of inorganic emitters. To extend their applications and improve the thickness, flexibility, and cost of commercial LEDs, intensive studies have been conducted to design and synthesize lighter, thinner, cheaper, and more flexible performant organic and organometallic emitters [51] that can potentially substitute LEDs [52]. Organic emitters can be also applied to lighting, i.e., the white organic light-emitting diodes, WOLEDs, that have been further investigated by some research groups [53]. The limitation of organic emitters as materials for devices is their decrease in emission power when they accumulate. The molecular aggregation-caused quenching (ACQ) of the emission can be seen in traditional organic emitters. Molecule emissions for OLEDs are often investigated in dilute solutions; however, they are utilized as a solid material such as a thin film [54]. The solid material is constituted of accumulated molecules; therefore, the ACQ is a relevant weakness of organic emitters for the fabrication of OLEDs.

Organic emitters are widely used as sensors for the analytical detection of biomolecules. There are biological structures and processes that cannot be probed or imaged by using intrinsic fluorescence due to endogenous fluorophores. In these cases, the biological structures must be labeled with exogenous fluorophores. The bio-probe-like behavior of these molecules offers a direct visualization of biological analytes at the molecular level and useful insights into complex biological structures and processes. The requirements for an emitting molecule to behave as a good exogenous fluorophore are the following: solubility in the biological medium; a specific association with the target molecules; high absorption intensity (I) and high emission quantum efficiency (Z), i.e., high cross-section; environmental stability; and the absence of photobleaching [55]. Organic emitters can be easily functionalized for binding to specific biological targets. Furthermore, organic synthesis allows for the designing of molecular structures that exhibit the desired emission frequency. The sensitivity of a bio-probe is determined by the brightness and contrast of its fluorescence before and after analyte binding. Biologic probes are utilized in biological media, where they are poorly soluble. Since aggregation often occurs in highly concentrated solutions, the ACQ effect limits the label-to-analyte ratio and forces researchers to use very diluted solutions of fluorophores; this limits the efficiency of the devices [56].

### 4.3. Conjugated Polymers as Contrast Agents for Biological Molecule Staining

**Figure 13:**
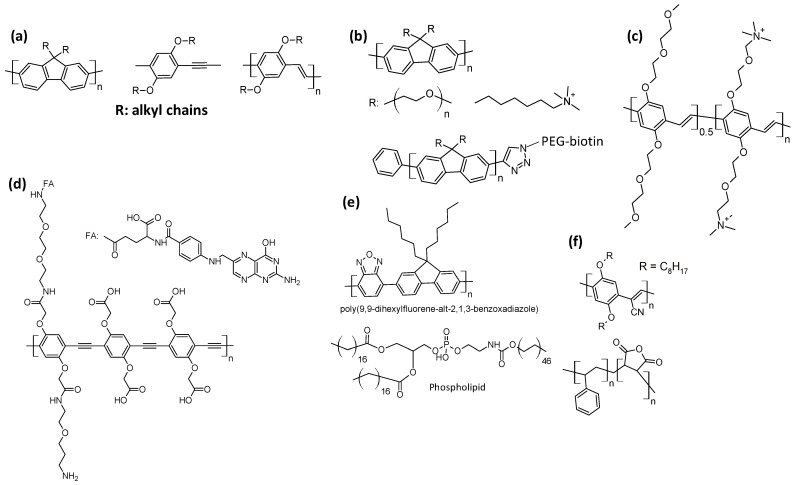
Representative polymeric structures of materials used for biodetection. (**a**) Conjugated polymers bearing alkyl chains and used in the form of Pdots [57,58]. (**b**) Polyfluorene functionalized with polyethylene glycol or alkyl ammonium chain and biotin [59]. (**c**) Polyethylene vinylene functionalized with diethylene glycol and butyl ammonium chains [60]. (**d**) Polyphenylene acetylene functionalized with folic acid, FA [61]. (**e**) Polydihexyl fluorene benzoxazole and phospholipid [62]. (**f**) Poly[2-methoxy-5-(2-ethylhexyloxy)-1,4-(1-cyanovinylene-1, 4-phenylene)] and poly(styrene-co-maleic anhydride) [63].

Due to their high absorption cross sections and emission quantum yields, conjugated polymers have been widely studied for the detection of biological molecules. Visible light-emitting polymers with high quantum yields, based on polyfluorene (blue emitter), polyphenylene ethynylene (green emitter), and polyphenylene-vinylene (yellow–green emitter), constituted pioneering materials studied for staining biological analytes. They were previously functionalized with alkyl chains for avoiding fluorescence quenching by self-assembling and were utilized in the form of Pdots or nanoparticles, as shown in Figure 13a [57,58]. Researchers also tried to functionalize these polymers to render them water-soluble and for binding specific biological analytes, as shown in Figure 13b–d. For example, polyfluorene was functionalized with polyethylene glycol and terminal PEG-biotin as a biological staining group [59]; with polyphenylene vinylene polymer types bearing ethylene glycol chains for water solubility and charged chains for interacting with phosphatyl serine residues and monitoring cell apoptosis [60]; and with polyphenylene ethynylene bearing charged chains for water solubility and folic acid (FA) for cancer cell recognition [61]. It is possible to prepare dots of a highly hydrophobic polymer and encapsulate them in hydrophilic nanovesicles, as displayed in Figure 13e,f; the hydrophilic section shown in Figure 13e consists of polyethylene glycol [62]. The encapsulating system can contain the functional group for recognizing the biological target; the orange-emitting poly[2-methoxy-5-(2-ethylhexyloxy)-1,4-(1-cyanovinylene-1,4-phenylene)] has been blended in a dot with poly(styrene-co-maleic anhydride), which allowed for coordination with the biological staining protein streptavidin, as shown in Figure 13f [63].

Due to the fact that biological tissues do not emit infrared light, staining materials capable of emitting infrared frequencies allow for imaging resolution improvement. This means that an organic IR emitter, when utilized as a contrast agent, allows for the selective detection of a biological analyte with a reduced noise/signal ratio. The emergence of near-infrared (NIR) imaging moved scientists to design conjugated molecules that emit frequencies in the infrared region; due to their high level of conjugation, conjugated polymers exhibit high fluorescence quantum yields and are well suited for this purpose. An effective strategy for preparing IR polymeric emitters consists of copolymerizing donor-conjugated with acceptor-conjugated molecules (copolymerization of D-π with A-π) or of polymerizing π-D-π-A-π molecules. Donor–acceptor conjugation is designed to ensure an absorption higher than 500 nm and a high probability of IR (>700 nm) emission. A representative example of a polymeric contrast agent NIR emitter was found in a work, where benzothiazole (acceptor) was conjugated with benzoxadiazole (donor) through fluorene units [64]. Alkyl-glycol-COOH chains avoid self-aggregation and ensure some hydrophilicity; the intramolecular FRET (Förster Resonance Energy Transfer, energy transferred from one chromophore to another one in proximity) benzoxazole–benzothiazole increases the fluorescence quantum yield. IR emitters for bioimaging with no hydrophilic chains have been prepared and arranged in nanoparticle dots; all of these contain fluorene with alkyl chains that are, in many cases, conjugated with thiophene, benzothiadiazole, pyrimidine–benzene fused rings, BODYPY (boron complexed to a dipyrromethane), and porphyrins containing Pt (II) as a metal ion [65,66,67,68]. A recent design consists of copolymerizing oligothiophenes (three units) as donors and pyrimidine as an acceptor for obtaining emission in the NIR-II [69].

**Figure 14:**
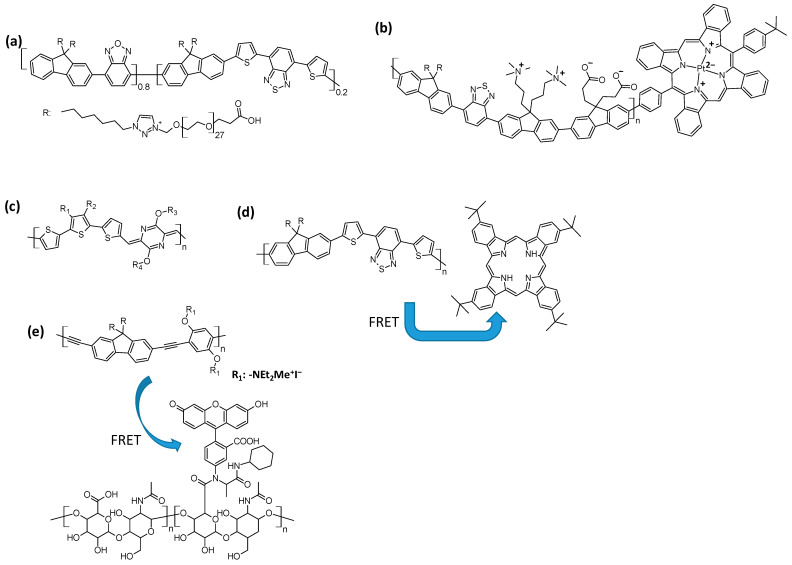
Structures of the polymers that exploit the FRET phenomenon [68,69,70,71]. (**a**) Water-soluble D-A polymer benzothiazole conjugated with a benzoxadiazole through fluorene units exhibiting internal FRET that enhances emission. (**b**) Fluorene conjugated with benzothiazole and bearing cell-recognizing groups and phthalocyanine complexed with Pt as FRET intramolecular acceptor. (**c**) D-A polymer thiophene pyrimidine. (**d**) Fluorene benzothiazole and intermolecular FRET with a phthalocyanine. (**e**) A fluorene phenyl acetylene copolymer and intermolecular FRET with a fluorescein linked with a saccharide.

A method for quenching or amplifying the fluorescence of conjugated polymers consists of coupling with a conjugated molecule co-blended in the same nanoparticle that interacts with the polymer. The copolymer fluorene–thiophene–benzothiadiazole can act as a light-harvesting material and transfer its emission to a co-encapsulated NIR porphyrin emitter [70]. Through FRET, the copolymer fluorene (with charged side chains)–phenylacetylene can transfer its fluorescence to polysaccharides bearing fluorescein, in which NIR emission is amplified (Figure 14) [71].

**Figure 15:**
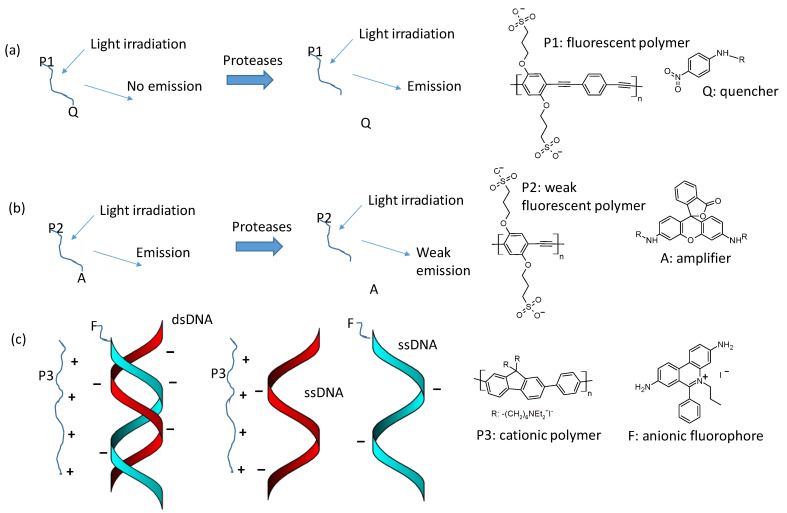
Description of ‘turn on’ and ‘turn off’ approach as described in the literature [72,73]: (**a**) Fluorescent polymer linked with a peptidic and ionic bond to a quencher; the interaction with a protease activates the polymer fluorescence by breaking the peptidic bond and removing the quencher (turn on). (**b**) Weak fluorescent polymer linked through a peptidic and ionic bond with an amplifier; the presence of the peptidase deactivates the amplifier effect by breaking the peptidic bond and removing the amplifier. (**c**) The cationic polymer P3 in water solution, in the presence of the double-strain DNA bonded with fluorophore, interacts with the ssDNA chain linked with fluorophore and increases its fluorescence by FRET. The same polymer in the solution with single-strain DNA chains interacts electrostatically and separately with the ssDNA chain with a fluorophore and with the other ssDNA chain; weak FRET can be observed.

The ‘turn off’ and ‘turn on’ approaches, as shown in Figure 15, take advantage of the phenomenon that a light-absorbing molecule absorbs the frequencies emitted by a molecule in close proximity. A conjugated emitting polymer can transfer the emitted light to a non-emitting molecule in close proximity, and this emission can be quenched. The conjugated emitting polymer can transfer its emission to a fluorescent compound, the emission of which is amplified (FRET phenomenon). An interesting application of this was found in protease detection [72]. The quencher or the amplifier was functionalized by esterification with a charged amino acid that interacted with the polymer. In the first case, irradiation at the polymer absorption generated no fluorescence because of the presence of a quencher; in the second case, the polymer absorption irradiation induced amplified emission from the amplifier. The proteases removed the bonding between the quencher or the amplifier with the amino acid. In the first case, the proteases induced the turning on of the polymer fluorescence; in the second case, the proteases induced the turning off of the amplifier. A similar approach has been used for detecting a single-strain DNA coupled with a complementary nucleic acid [73]. This was linked with a cationic fluorophore and kept in contact with a fluorescent cationic polymer. In the presence of the complementary DNA chain, the FRET from the polymer to the fluorophore amplified the fluorophore’s fluorescence; in the presence of a non-complementary chain, the amplification was weak because the two chains competed or interacted with the polymer.

**Figure 16:**
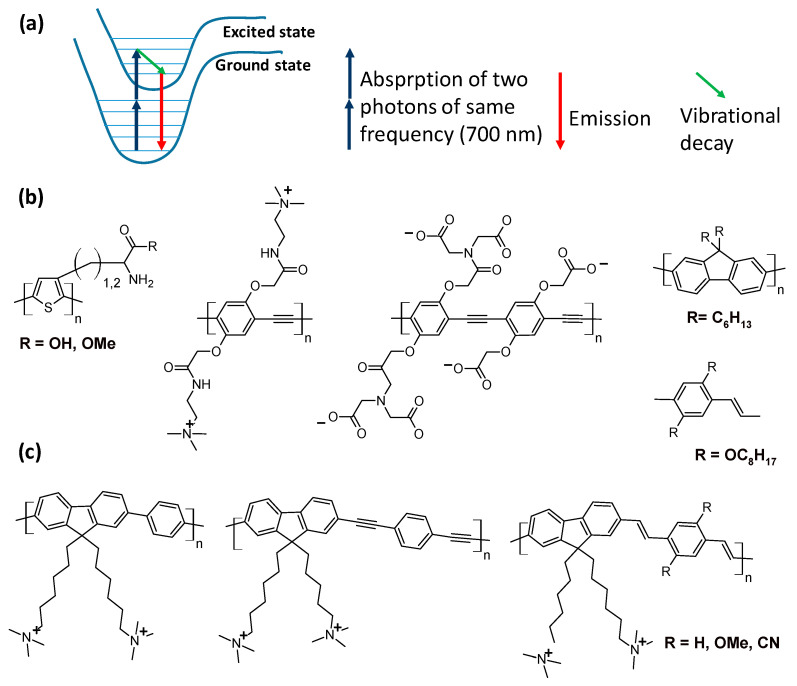
Representative polymeric structures of polymers used in two-photon spectroscopy [74]. (**a**) Principle of two-photon spectroscopy; the absorption wavelength is the sum of two photon wavelengths. (**b**) Polythiophene and polyphenyleneacetylene functionalized with positively and negatively charged groups with affinity for live mouse fibroblast cells; polyfluorene and polyphenylene vinylene functionalized with alkyl chains used for fluorescent nanoparticles. (**c**) Polyfluorene phenylene, polyfluorene acetylene, and polyfluorene phenylene vinylene functionalized with alkyl ammonium chain for interaction with nucleic acids.

Further progress consisted of two-photon absorption microscopy (TPA), which bases its operation on emitters that absorb two photons to reach the absorption frequency. In this analytical technique, the absorption frequency falls in the infrared, and the resolution is further improved. TPA is favored in compounds with a high level of conjugation, giving them high cross-section values; for this reason, conjugated polymers fit better than small molecules for this technology [74]. Polyacetylenes, poly(p-arylenevinylenes, p-phenylene, polythiophenes, polybenzobisazoles, polyquinolines, polyquinoxalines, polyantrazolines, and polyfluorenes have been investigated as TPA materials upon functionalization for cell or biological tissue staining, as shown in Figure 16.

**Figure 17:**
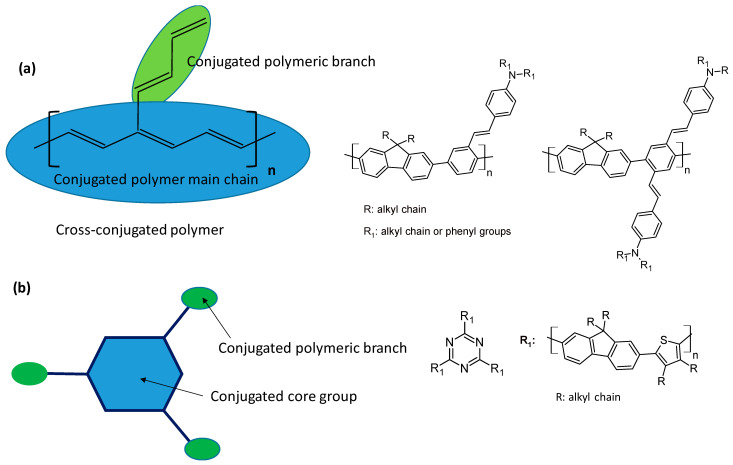
Description of the design for improving the conjugation extension and having more efficiency in two-photon absorption [74]. (**a**) A fluorophore design for two-photon spectroscopy by extending the conjugation on branch units. (**b**) Design of conjugated polymers for two-photon spectroscopy based on a conjugated core unit linked to conjugated polymeric branches.

These polymeric platforms have also been optimized for improving fluorescence upon two-photon absorption by increasing their cross-section values. Large conjugated monomers were designed to ensure a high possibility of efficient two-photon absorption. The strategies consisted of preparing cross-conjugated polymers using conjugated polymers in which the conjugation extends along the polymer branches or star-shaped compounds, where a conjugated core group is connected with conjugated polymeric branches. The polymers, which were not water-soluble, displayed effective high TPA efficiency and were utilized in the form of nanoparticles, as shown in Figure 17.

### 4.4. Novel Emissive Compounds for Limiting Fluorescence Quenching Due to Molecular Aggregation

In 2001, Tang and coworkers discovered that emitters, with their propeller-shaped rotor-like structures, when aggregated, increase fluorescence [75]. Conformations are characterized by the low-frequency torsional motions of isolated molecules, and they emit very weakly in solutions. They show strong fluorescence in the aggregates, mainly due to the restriction of their intramolecular rotations. This property is called AIE (aggregation-induced emission). The modification of the first molecules with AIE properties that were synthesized by Tang allowed for the synthesis of different emitting compounds for a wide range of applications [54,76]. Taking advantage of the phenomenon of AIE, organic light-emitting diodes have been fabricated to offer both primary RGB (red, green, and blue) colors and white-light emission [54,76,77,78]. This is because AIE molecules can exhibit fluorescence quantum yields up to unity in the solid state, with emission colors covering the whole visible and even NIR range [54]. Liquid crystal displays (LCDs) can also potentially take advantage of AIE. Current liquid crystal display devices require back-lighting, and as a consequence, their mechanical design is complicated and energy-consuming. Liquid crystals based on AIE molecules are, themselves, emissive; they do not require back-illumination for screen displays [76].

The same molecules, properly functionalized for binding to bioanalytes, were employed as bio-probes. AIE molecule applications range from optoelectronics to bioanalysis, as recently noted [79].

The mechanisms of the AIE phenomenon have been studied in recent years; they allow us to understand the many aspects of emission. Traditional organic emitters are planar push–pull molecules, where a donor group is connected to an acceptor through a π-bridge (D-π-A). When the molecule is photo-excited, a charge separation is induced. Decaying to the ground state transfers the electrons in the opposite direction with respect to the charge separation of the excited state. The planar shape of the molecule is not altered in the processes of absorption and emission and the intramolecular charge transfer is known as planar intramolecular charge transfer (PICT). In the aggregated phase, the excited planar molecules with charge separation self-assemble in a centrosymmetric supramolecular structure that weakens the charge separation state and inhibits emission. The first molecular systems that evidenced AIE were propeller-like molecules that did not show polarity. The phenomenon was rationalized with the theory of restricted rotations in the condensed phase [54,75,80]. In a dilute solution, free intramolecular rotation generates vibrational relaxation channels for the decay of the excited state that do not generate emissions. The aggregation induces the slowing or stopping of this rotation, and molecular luminescence is generated. Molecules like 1,1,2,3,4,5-hexaphenylsilole (HPS) and tetraphenylethylene (TPE) contain rotating units such as phenyl rings. Rotor-containing fluorogens undergo low-frequency motions in dilute solutions, causing the roto-vibrational decay of the excited states that makes the fluorogens non-emissive. In the aggregates, these motions are blocked by intermolecular steric interaction, which obstructs the electronic transition through vibrational decay and opens the emitting pathway. Push–pull molecules can also exhibit AIE properties. In the excited state, a D-π-A compound that is not rigidly planar can exhibit a stable twisted shape with charge separation. Ground-state restoration occurs with a twisted intramolecular charge transfer (TICT) mechanism. The emission in the condensed phase depends on the modality of aggregation. 4-Diethylamino-2-benzylidene malonic acid dimethyl ester, while possessing very low quantum yields in a solution and in the amorphous phase, is an excellent emitter in the crystalline solid state; as a consequence of its restricted rotation and dimeric J aggregation, quantum yields approached 0.5 [81]. It is known that J aggregation improves and moves at higher emission wavelengths with respect to the monomer [82]. In the crystalline phase, the molecule showed a further enhanced emission, showing the phenomenon called CIE (crystallization-induced emission). More accurate studies showed that the viscosity of the solvent slows down the torsional relaxation of the organic compound, determining a higher photoluminescent quantum yield [83]. In another work, the effect of an increasing conjugation between the two donor and acceptor ends of the chromophores was investigated. The result was that chromophores with greater conjugation aggregated in herring-bone assemblies behave like AQE molecules [84]. A further modification was the increase in the electron-withdrawing character of the acceptor group. The molecule enhances emission in the condensed phase and also in ‘rigid environments’. Studies in different solvents, with different viscosities, highlighted the strong dependence of the efficiency of the TICT mechanism on the solvent viscosity and the presence of long-life optical gain from the first excited state in viscous solvents [85].

Molecules with propeller-shaped structures, endowed with strong donor and acceptor functional groups, exhibit AIE behavior, and the process of emission involves contemporaneous RIR and TICT mechanisms [86].

The behavior of propeller-shaped molecules as biological sensors, when endowed with a binding group of biological molecules, can be explained by the change in emitting properties when the rigidity of the environment is modified [87]. In the absence of the biological molecule in the medium, the compounds did not emit light. The emission was measurable when the sensor was bound to the biological functionality. The connection with the biological molecule limits the roto-vibrational freedom of the sensor and favors emission.

A further application of the emitters in nanomedicine is in theranostics. According to Warner’s definition, theranostics is a portmanteau of therapeutic and diagnostics [88]. A single molecular platform includes a therapeutic and diagnostic agent. The diagnostic agent works as the marker of the sick cells and delivers the drug. The diagnostic molecule is a fluorescent molecular unit that accumulates inside the target cells, carrying the therapeutic active unit. The fluorescence allows for a visualization of the cells, where the theranostic agent is accumulated. The changes in fluorescence with time allow specialists to follow the activity of the therapeutic agent.

Theranostic nanoparticles are mainly studied for cancer treatment because of the limitations of chemotherapy [89]. Conventional anticancer therapeutic drugs are non-selective. Most of the drugs also alter the metabolism of normal cells. Thus, to deliver the drugs to the target cells while avoiding normal cells, inorganic nanoparticles were pioneering materials applied in drug delivery and as contrast agents to detect cancerous cells [89,90,91]. Lipids, polysaccharides, peptides, and synthetic polymers were successively functionalized as drug carriers and disease markers because of their biocompatibility, flexibility, and easy modification [92]. In recent years, after the discovery of the AIE phenomenon, researchers have also started to utilize small emitting molecules as platforms for diagnosis and drug delivery. Binary systems in which the porphyrin is linked to an AIE molecule were described for the first time in 2012 [93]. The AIE unit aggregates in the cancer cells and emits light. When the system is irradiated at a specific frequency, the dye transfers energy to the porphyrin by fluorescent resonance energy transfer (FRET). As a consequence of the energy transfer, porphyrin generates singlet oxygen, which kills the cancer cells [94]. The AIE molecule works as a diagnostic agent and also as a drug carrier. The porphyrin is the therapeutic agent. The system was synthesized with different AIE molecules [95,96,97,98,99,100]. Binary systems where AIE molecules were connected to a therapeutic agent that is different from porphyrin were also synthesized and tested [97,101,102,103].

**Figure 18:**
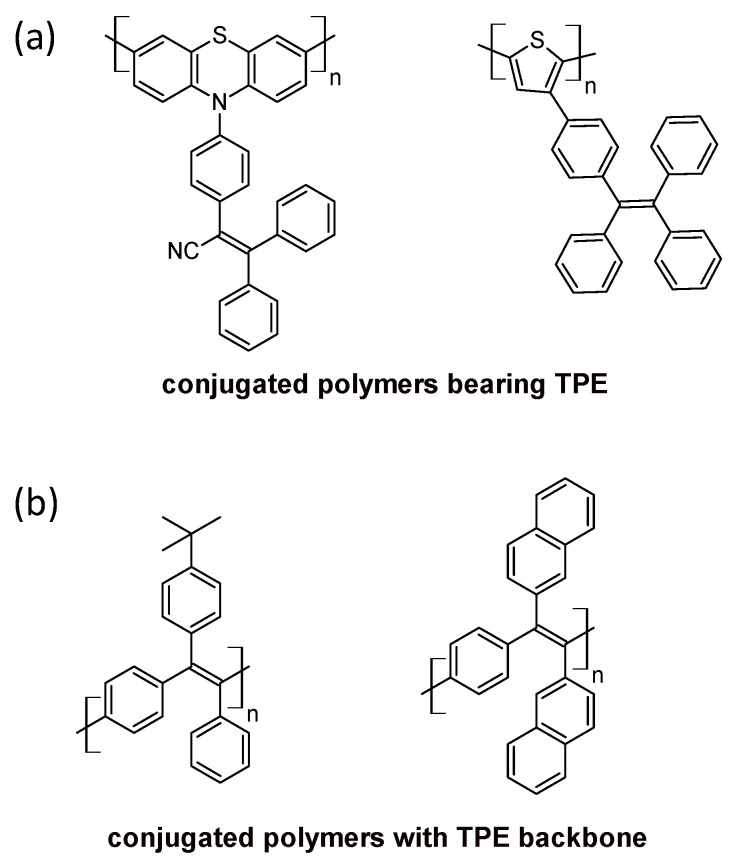
Some structures of AIE polymers from the literature [104,105]. (**a**) Polymeric backbones made of non-AIE monomers phenothiazine or thiophene endowed with AIE triphenyl ethene and tetraphenyl ethene, respectively. (**b**) Polymers constituting AIE monomers triphenyl ethene and tetraphenyl ethene.

AIE in polymers. The AIE phenomenon in conjugated polymers is a recent topic, which has developed since 2016, due to the fact that polymers tend to self-aggregate and quench fluorescence [104]. Compared with small molecules, AIE polymeric materials display the highest mechanical strength and highest fluorescence (because of the presence of many fluorophore functional groups, better processability, and the possibility of fabrication into large-area thin films [105]). One strategy for preparing AIE polymers consists of building polymers from AIE monomers. A conjugated polymer, constructed with no AIE monomers like polythiophene or polyphenothiazine, can be endowed with AIE small molecules like tetraphenylethylene (Figure 18a). A second strategy consists of preparing conjugated polymers with an AIE tetraphenylethylene backbone (Figure 18b) [104,105].

## 5. Addressing Degradation in Photonic Biomaterial

Organic solar cells are gaining attention for their potential to provide flexible, lightweight, and environmentally friendly alternatives to traditional photovoltaic technologies. However, a significant drawback is their tendency to degrade over time, compromising their efficiency and reliability. This degradation can result from various factors, including exposure to oxygen, moisture, UV light, and thermal stress [106].

These elements cause changes at the molecular level, leading to reduced performance and shorter lifespans for organic solar cells. To mitigate this issue, researchers are exploring several workarounds. Strategies such as encapsulation [107,108] and the development of more stable organic compounds can help improve the longevity of organic solar cells [109], making them more viable for technological applications. These approaches focus on creating robust protection around the sensitive components within organic solar cells, thereby reducing the risk of degradation from environmental stressors like oxygen, moisture, and UV light. By forming a barrier, these strategies can significantly reduce the rate of deterioration, helping the solar cells maintain their efficiency and performance over time. Additionally, some approaches aim to enhance the intrinsic stability of the organic materials used in the cells. This can involve chemical modifications or the introduction of stabilizing additives [110] that strengthen the material’s resistance to heat, light, and oxidation.

The ultimate goal is to increase the durability of organic solar cells, making them more viable for long-term use in various applications [111]. As these cells become more resilient, they can be integrated into a broader range of technologies, providing a more sustainable and flexible alternative to traditional photovoltaic systems. By addressing these degradation issues, researchers hope to unlock the full potential of organic solar cells, contributing to a more sustainable energy future.

## 6. Considerations of the State of the Art in Research and Conclusions

### 6.1. Organic Photovoltaic Technology

Due to their high efficiency in converting solar light into energy, the advent of the perovskite solar cell shifted research interests away from OPV and DSSC devices. Research on OPV materials persists in the field of biomedicine when perovskites cannot meet the needs of biocompatible biomaterials. Currently, conjugated polymers constitute the most studied OPV materials for biomedical applications. DSSC technology has not been studied in the context of biomaterial devices because the development of biocompatible organic compounds as hole transporters still cannot sufficiently replace inorganic materials. To render devices biocompatible and to revive interest in this field of research, conjugated polymers as hole transporters seem to be the best alternative to iodine couples or transition metal compounds.

### 6.2. Organic Emitting Materials

OLED technology has been extensively developed; devices based on these materials have thus gained an important place in the market. For this reason, research on new emitting materials for electronics has slowed down in recent years. Research is still active in the field of biodetection, which requires new biocompatible emitting materials to improve the resolution of bioimaging technology. In this field, conjugated polymers and small molecules are both under investigation.

### 6.3. Future Perspectives

Research on the biophotonic applications of conjugated polymers can find new perspectives by investigating new chemical structures, different from the ones shown in Figure 2. Research into classical conjugated polymers seems well developed, and it is difficult to imagine a strong breakthrough in the future. Light energy conversion mechanisms can be different depending on the chemical structures of the compounds. The development of new conjugated small molecules can inspire researchers of new conjugated polymers. As shown in Section 4, AIE-conjugated molecules inspired the preparation of AIE-conjugated polymers, the structures of which differ from the structures shown in Figure 2. As an example, in recent years, environmentally sensitive emitting small molecules have been prepared and studied, the structures of which can inspire the preparation of environmentally sensitive conjugated polymers that can, in turn, significantly improve the resolution of bioimaging technology [112].

In conclusion, conjugated polymers have demonstrated their versatility and significance across a spectrum of applications, spanning from photonic devices to biomedical advancements. Their unique properties, such as tunable optical characteristics and biocompatibility, have enabled their use in myriad cutting-edge technologies. From optoelectronic devices to photodynamic therapy for combating pathogens, conjugated polymers continue to pave the way for innovation in both research and practical applications. As technology advances and our understanding of these materials deepens, we can expect further breakthroughs and the continued integration of conjugated polymers into diverse fields, ultimately driving progress in both photonics and biomedicine.

## Data Availability

Not applicable.
